# Endurance Exercise as an “Endogenous” Neuro-enhancement Strategy to Facilitate Motor Learning

**DOI:** 10.3389/fnhum.2015.00692

**Published:** 2015-12-22

**Authors:** Marco Taubert, Arno Villringer, Nico Lehmann

**Affiliations:** ^1^Department of Neurology, Max Planck Institute for Human Cognitive and Brain Sciences, LeipzigGermany; ^2^Clinic for Cognitive Neurology, University Hospital Leipzig, LeipzigGermany

**Keywords:** neuromodulation, endurance exercise, motor learning, brain, neuroplasticity, lactate, motor cortex, acute

## Abstract

Endurance exercise improves cardiovascular and musculoskeletal function and may also increase the information processing capacities of the brain. Animal and human research from the past decade demonstrated widespread exercise effects on brain structure and function at the systems-, cellular-, and molecular level of brain organization. These neurobiological mechanisms may explain the well-established positive influence of exercise on performance in various behavioral domains but also its contribution to improved skill learning and neuroplasticity. With respect to the latter, only few empirical and theoretical studies are available to date. The aim of this review is (i) to summarize the existing neurobiological and behavioral evidence arguing for endurance exercise-induced improvements in motor learning and (ii) to develop hypotheses about the mechanistic link between exercise and improved learning. We identify major knowledge gaps that need to be addressed by future research projects to advance our understanding of how exercise should be organized to optimize motor learning.

## Introduction

The optimization of motor learning is of particular relevance in many sport-related settings such as competitive sports, disease prevention, rehabilitation after neurological or orthopedic injury as well as physical education. A huge body of literature in movement and training science proposes strategies to optimize motor skill learning with a strong emphasis on practice distribution (for example massed vs. distributed practice), scheduling (blocked vs. random practice), variation of motor tasks (constant vs. variable practice) as well as movement feedback or attentional focus (Magill, 2011; Schmidt and Lee, 2014).

From a more mechanistic perspective, strategies to improve motor learning may benefit from a deeper understanding of the underlying neurobiological mechanisms of skill acquisition, stabilization and retention in the brain. Thereby, targeted strategies can be developed to specifically modulate learning-related mechanisms with the aim to augment motor learning.

For example, transcranial electric or magnetic stimulation can be used to modulate brain function and behavior through external application of weak electric currents or magnetic fields throughout the scalp (Nitsche and Paulus, 2000; Reis et al., 2008; Dayan et al., 2013). One widely used technique is transcranial direct current stimulation (tDCS). TDCS of the primary motor cortex (M1) has been shown to increase long-term potentiation-like (LTP-like) plasticity or improve motor memory retention (Reis et al., 2008, 2009). Such external stimulation techniques allow for focal modulation of cortical excitability and offer intriguing possibilities for example in stroke rehabilitation (Nowak et al., 2009).

Here we propose physical exercise as a more “endogenous” neuromodulation strategy to improve motor learning and brain plasticity. Mounting evidence demonstrates that physical exercise affects brain structure and function from the molecular to the systems level of brain organization (Voss et al., 2013b). Physical exercise facilitates long-term potentiation (LTP)-like plasticity in M1 (Singh et al., 2014b) and increases the level of learning-related neurotrophins (Rojas Vega et al., 2006). These and other mechanisms of physical exercise are discussed to potentially modulate motor learning (Fabel et al., 2009; McHughen et al., 2010; Cantarero et al., 2013).

Roig et al. (2013) provided a comprehensive summary about the behavioral effects of exercise on declarative and procedural memory processing. Here, we will focus on motor learning and review, in particular, the existing neurobiological evidence to generate hypotheses about the causal relationship between exercise and online/offline motor learning. We acknowledge that physical exercise may modulate motor performance via processes outside the central nervous system such as increases in muscle strength and flexibility (Schmidt and Lee, 2014). While we did not ignore these peripheral sources, we believe that modulation of brain function and structure through exercise constitutes largely unexplored mechanisms to optimize motor learning. Physical exercise is a natural and “endogenous” neuro-enhancement strategy potentially relevant for disease prevention, rehabilitation and education. To understand how exercise may enhance motor learning and neuroplasticity, it is important to characterize the neural correlates of motor learning.

## Online and Offline Motor Learning

Online motor learning is expressed as gain or loss in motor performance within a motor practice session (motor memory acquisition) while offline motor learning reflects performance changes between subsequent practice sessions (Dayan and Cohen, 2011). Hence, offline learning is thought to depend also on neuroplasticity within the period after task performance (Muellbacher et al., 2002). Subjects may reside in the resting-state, sleep or perform other tasks in this period which may positively or negatively influence offline learning-related neuroplasticity. For example, studies have shown abolished offline learning (motor memory consolidation and retention) through learning an interfering motor task (negative transfer, Brashers-Krug et al., 1996) or enhanced offline learning through sleep (positive transfer, Walker et al., 2003).

Here we propose that physical exercise induces positive transfer effects on online and offline motor learning through facilitation of the underlying neural processes of motor memory acquisition, consolidation and retention. This implies that certain aspects of exercise-induced brain changes are causally linked to the performance gains seen during online and offline periods as well as its neurobiological correlates in the brain. At present, this causal link has not yet been established and our hypothesis is based on independent evidence from (i) behavioral studies showing positive effects of exercise on motor learning as well as (ii) neurobiological studies on exercise-induced brain changes. Therefore, we will first review these separate pieces of behavioral and neurobiological evidence before hypotheses are generated about the causal link between exercise, motor learning and their underlying mechanisms.

After reviewing the neurobiological evidence, we will highlight behavioral studies showing improved motor learning through physical exercise scheduled before (acute or habitual exercise) or after (post-practice period) a motor practice session.

## Neural Correlates of Motor Learning

We will now briefly highlight some of the existing evidence on motor learning-related changes in the brain. With this, we would like to inform readers about relevant brain changes that are important for a better understanding of the effects of exercise on motor learning. For a more detailed review on the neural correlates of motor learning, the reader is referred to review articles by Doyon and Benali (2005), Monfils et al. (2005), and Dayan and Cohen (2011).

In brief, both online- and offline motor learning are associated with distinct changes in brain activation in typical sensorimotor networks (e.g., motor cortex, basal ganglia, cerebellum) and higher-order associative networks (e.g., prefrontal, parietal and temporal cortices). Online motor learning in the early practice period engages prefrontal, parietal and partly hippocampal brain regions in addition to sensorimotor cortical-striato-cerebellar networks (Karni et al., 1995; Honda et al., 1998; Floyer-Lea and Matthews, 2005; Albouy et al., 2008) while the prefrontal contributions decrease in the later practice period (Poldrack et al., 2005) and the motor memory seems to be stabilized in sensorimotor cortical and subcortical (striatum and cerebellum) networks. More extensive periods of motor practice induce structural changes in cortical gray matter and underlying white matter tracts (Scholz et al., 2009).

The structure of white matter fiber tracts regulate the timing and speed of action potentials across axons which are critical for the occurrence of learning-related neuronal plasticity (“neurons that fire together, wire together”) (McKenzie et al., 2014). Training-induced plasticity in M1 may occur through lasting modulations in synaptic transmission (Butefisch et al., 2000; Rioult-Pedotti et al., 2000; Donchin et al., 2002; Antonov et al., 2003) including synaptogenesis (Xu et al., 2009) and the coordinated strengthening (e.g., LTP) and weakening (e.g., LTD) of synaptic connections (Mayford et al., 2012; Lee et al., 2014). In this respect, LTP and long-term depression (LTD) are considered as the cellular analog of motor learning (Rioult-Pedotti et al., 2000; McConnell et al., 2009; Cantarero et al., 2013). LTP and LTD reflect sustained changes in synaptic efficacy in response to the correlated arrival of action potentials between neurons (Hebb’s learning rule, “neurons that fire together, wire together,” Hebb, 1949). In humans, neurophysiological studies showed that motor learning (i) requires LTP-like plasticity in M1 (Cantarero et al., 2013), (ii) increases the size of the movement representation of trained limbs in M1 (Pascual-Leone et al., 2005) and (iii) enhances motor corticospinal excitability (Muellbacher et al., 2002), although the relationship between cortico-spinal excitability and motor learning is complex (Tunovic et al., 2014).

In animals, LTP induction is linked to cellular structural changes (Toni et al., 1999; Harms et al., 2008). These structural changes rely on *de novo* protein synthesis (Lu et al., 2008) and injecting protein synthesis inhibitors in M1 results in a loss of previously learned skills as well as an impairment in new motor skill acquisition (Kleim et al., 2003). Worsened motor skill learning was correlated with reduced synapse number and size (Kleim et al., 2003) and learning a new motor skill rapidly increases the number of new synaptic spines in M1 (Xu et al., 2009). While the overall spine density returns to initial values soon, the newly formed spines are preferentially stabilized through subsequent practice and outlast the end of the training period (Xu et al., 2009). Further studies reported synaptogenesis after a few weeks of motor learning (Black et al., 1990; Kleim et al., 2002b) that was specific to the cortical representation of the trained limb and accompanied by an increase of motor map size (Kleim et al., 2002b, 2004). Such changes were not observable in the untrained limb representation and occur as a consequence of skilled motor activity instead of repetitive limb use (Kleim et al., 1996; Plautz et al., 2000; Tyč et al., 2005) or even strength training (Remple et al., 2001; Jensen et al., 2005). The prevailing and generally accepted view is that motor learning reorganizes neuronal and synaptic connections, whereas endurance exercise mainly influences the supportive vascular components (Churchill et al., 2002).

At the molecular level, motor learning reduces the concentration of the inhibitory neurotransmitter *γ-aminobutyric acid* (GABA) in M1 (Floyer-Lea et al., 2006; Stagg et al., 2011). Furthermore, the neurotrophic factor BDNF (*brain-derived neurotrophic factor*) is linked to functional plasticity in the human motor system. Subjects carrying the Val66Met polymorphism of the BDNF gene, which is known to affect activity-induced BDNF secretion (Egan et al., 2003), show reduced corticospinal excitability and reduced motor map reorganization in response to motor learning (Kleim et al., 2006). The Val66Met polymorphism also negatively affects online and offline learning of a complex motor tracking task (McHughen et al., 2010) but had no effect on learning a serial reaction time task (Freundlieb et al., 2012; Morin-Moncet et al., 2014). In Mice with BDNF mutations show diminished responses to excitability-enhancing brain stimulation of M1 (Fritsch et al., 2010). Not last, the loss or critically low levels of BDNF are associated with motor system dysfunction, for example with severe neurodegenerative diseases (Teixeira et al., 2010; He et al., 2013).

The central question that runs through this article is how endurance exercise influences these motor learning-induced brain changes at the systems-, cellular, and molecular level to create a productive neural environment for neural plasticity during online and offline periods of motor learning.

## Neural Correlates of Exercise

Like motor learning, physical exercise elicits neural changes from the systems- to the molecular level of brain organization. We will restrict the following review to exercise-induced brain changes that are potentially important to influence motor learning-induced neuroplasticity.

### Systems-Level

To investigate exercise-effects at the *systems-level*, research in humans was performed using, e.g., transcranial magnetic stimulation (TMS) or magnetic resonance imaging (MRI). For TMS and functional MRI (fMRI), exercise-induced changes were found for corticospinal excitability, LTP-like plasticity and functional connectivity immediately or some minutes after the exercise interventions while structural MRI studies assessed lasting changes in gray and white matter after weeks to months of exercise. Such acute and lasting effects may contribute differently to improvements in online and offline motor learning.

#### TMS and fMRI

Transcranial magnetic stimulation is a non-invasive technique to focally stimulate superficial cortical brain regions across the scalp. Application of a single, suprathreshold TMS pulse over the primary motor cortex (M1) activates peripheral target muscles that can be recorded via electromyography. This response is referred to as motor evoked potential (MEP). The most commonly used TMS measures that characterize motor learning-related changes are (i) the size of cortical area from which an MEP could be evoked (movement representation), (ii) the lowest stimulus intensity to evoke an MEP (motor threshold) and (iii) the size of the MEP at a defined stimulation intensity (1 mV MEP). In general, motor learning increases motor map size, decreases the motor threshold, and increases MEP amplitudes (Pascual-Leone et al., 2005; Adkins et al., 2006). More recently, these indices have also been recorded in response to endurance exercise. Exhaustive exercise lowers the motor threshold indicating increased cortico-spinal excitability (Coco et al., 2010). Also, increased cortical excitability was found in very active compared to sedentary subjects (Cirillo et al., 2009). More recent studies, however, were not able to replicate the exercise-induced increase in cortico-spinal excitability and instead found evidence that exercise enhanced neuroplasticity in M1 (please see below McDonnell et al., 2013; Mang et al., 2014). Paired pulse TMS (ppTMS) allows to specifically examine local inhibitory and facilitative mechanisms within M1 (intra-hemispheric excitability) as well as between M1 and distant brain regions in the ipsi- and contralateral hemisphere (intra- and interhemispheric excitability). PpTMS pairs two TMS pulses over M1 with particular inter-stimulus intervals to target inhibitory (5 ms or less) or facilitatory (10–25 ms) mechanisms. A decrease in intracortical inhibition, which seems to be dependent on the level of the inhibiting neurotransmitter GABA, is generally assumed to reflect a favorable environment for the induction of neuroplasticity and therefore motor skill learning (Singh and Staines, 2015). Reductions in local GABA concentrations in M1 are correlated with motor learning in a serial-reaction time task (Stagg et al., 2011), a sensorimotor adaptation paradigm (Kim et al., 2014) as well as a motor tracking task (Floyer-Lea et al., 2006). Using acute bouts of exercise, Yamaguchi et al. (2012) reported a decrease in short-interval intracortical inhibition (SICI) of the leg area (tibialis anterior and soleus muscles) after just 7 min of low-intensity cycling (Yamaguchi et al., 2012). Similar effects were observed for the upper extremity (first dorsal interosseous muscle) after 30 min of low–moderate or moderate–high intensity cycling (Smith et al., 2014). Likewise, 20 or 30 min of continuous biking with moderate intensity decreased SICI measured in the extensor carpi radialis and abductor policis brevis muscles (Singh et al., 2014a; Snow, 2014). Moreover, a rodent study showed that exercise upregulates genes associated with the excitatory glutamatergic system and downregulates genes related to the inhibitory GABA system in the hippocampus (Molteni et al., 2002). Taken together, these studies provide evidence that exercise at low, moderate or even high intensities rapidly reduces intracortical inhibition and that this effect is not limited to the exercised limbs. This may be beneficial for online motor learning. It must be mentioned that an increased intracortical inhibition in lower extremity M1 representations (vastus lateralis muscle) was observed during fatiguing cycling exercise (Sidhu et al., 2013) indicating that exercise at very high intensities may attenuate learning if motor practice involves similar effectors.

Finally, more recent studies examined the effect of endurance exercise on TMS protocols aiming to experimentally induce changes in synaptic efficacy using paired-associative stimulation (PAS; LTP- or LTD-like plasticity). Basically, this technique induces cortical LTP-like plasticity by first stimulating a peripheral nerve electrically, followed by a TMS pulse of the corresponding M1 area several milliseconds later. This enables researchers to study synaptic plasticity *in vivo* and to reduce the influence of numerous boundary conditions normally affecting behavior and associated brain changes (e.g., inter-individual differences in motor learning). Cirillo et al. (2009) tested the effect of regular physical activity on PAS-evoked neuroplasticity. Participants were divided into two groups dependent on self-reported physical activity level. The sedentary group performed physical activity less than 20 min per day on no more than 3 days per week, whereas the active group performed moderate-to-vigorous aerobic activity more than 150 min per day on at least 5 days per week. Active subjects showed increased LTP-like plasticity, as measured by the MEP amplitude of the abductor pollicis brevis (APB) muscle (hand muscle). Notably, similar effects were also registered in other experiments focusing on the effects of a single bout of exercise. For example, enhanced LTP-like plasticity in the APB muscle was observed after 20 min of moderate-intensity cycling (Singh et al., 2014b). This beneficial effect applies for higher exercise intensities as well, since PAS-effects were also noted after 20 min of high-intensity interval cycling (Mang et al., 2014). However, LTP-like plasticity was not enhanced in the soleus muscle (lower extremity) of endurance athletes but pronounced in skill athletes (Kumpulainen et al., 2015). The reason for the diminished plasticity in lower limbs and the enhanced plasticity in upper limbs in endurance athletes remain speculative.

Beyond M1, acute exercise has been shown to change large-scale brain network connectivity in the resting-state. Rajab et al. (2014) compared 20 min of moderate-intensity exercise (70% of age-predicted HR_max_) with a resting control group (*n* = 15). Functional connectivity was tested before and immediately after the exercise bout and increased connectivity was found in sensorimotor and thalamic-caudate networks (Rajab et al., 2014).

To sum up, acute exercise induces facilitative effects on early neuroplasticity (within the first hour after exercise). However, the dose-response relationship between exercise parameters, especially exercise intensity, and TMS indices is not clear to date (Singh and Staines, 2015) and long-term intervention studies on corticospinal excitability or PAS-induced plasticity are still missing.

#### Structural MRI

An excellent method to observe brain morphology changes in humans is MRI. MRI can be used to non-invasively assess the shape and size of brain regions and to compare these measures between participants and across time within single individuals. Morphological measures such as gray matter volume/density or cortical thickness are derived from segmentation of individual brain images into distinct tissue classes (e.g., gray matter, white matter and cerebrospinal fluid). In recent years, a considerable amount of studies demonstrated structural changes in, e.g., gray matter density after complex motor learning (Draganski et al., 2004; Taubert et al., 2010). The cellular correlates of gray matter changes observed with MRI are still unknown and recent studies combining MRI with histological assessment in animals provide new insights into how MRI changes are correlated with alterations at the cellular level (Lerch et al., 2011; Hamaide et al., 2015).

First, cross-sectional studies in humans have found associations between brain structure and motor behavior. Schlaffke et al. (2014) directly compared grey matter density (GMD) between long-distance endurance athletes, martial artists and a non-sport control group not reporting participation in any regular physical activities. The idea of comparing endurance vs. martial artists is based on their differing metabolic profile (mainly aerobic vs. mainly anaerobic). In comparison to controls, statistical analysis of GMD across the whole brain showed higher GMD in the supplementary motor area/dorsal premotor cortex (BA 6) in both athlete groups. Endurance athletes additionally revealed higher GMD in medial temporal lobe. The authors conclude that structural differences in these regions in the athlete groups may be related to motor control and motor skill acquisition (Dayan and Cohen, 2011; Tomassini et al., 2011).

Longitudinal studies with repeated MRI measurements before and after training provide further insight into potential causes of brain differences since the aforementioned variations in gray matter may be attributed to physical activities or alternative factors such as genetic predispositions. In a longitudinal study with elderly humans, aerobic exercise for 1 year reversed the age-related decline in gray matter and increased hippocampal volume of approximately 2% (Erickson et al., 2011). Besides the hippocampus, the prefrontal cortex is also vulnerable for exercise-induced gray matter changes in elderly humans (Colcombe et al., 2006). Both the hippocampus and the prefrontal cortex are relevant for learning, memory and cognitive control and the fMRI literature on motor learning suggests that both brain regions are involved in the early period of motor skill learning (please see Neural Correlates of Motor Learning). Also, motor learning induces structural GMD changes in the hippocampus and prefrontal cortex (Boyke et al., 2008; Taubert et al., 2010; Sehm et al., 2014). Therefore, long-term exercise may exert beneficial effects on motor learning by priming brain regions implicated in motor skill acquisition such as the hippocampus and/or prefrontal cortex.

With respect to motor learning, however, the aforementioned MRI studies have tested exercise effects using relatively long observation periods (6–12 months). Interestingly, two MRI studies with rodents (Sumiyoshi et al., 2014; Cahill et al., 2015) recently demonstrated that exercise over 1–4 weeks affects brain regions well known to be involved in motor function and learning (please see Neural Correlates of Motor Learning). In one of these studies, Cahill et al. (2015) exposed male mice to 4 weeks of voluntary exercise and compared alterations in brain structure to inactive controls using high resolution MRI. The authors registered exercise-induced gray matter changes in several brain structures, amongst them hippocampus, dentate gyrus, stratum granulosum of the dentate gyrus, cingulate cortex, olivary complex, inferior cerebellar peduncle and regions of the cerebellum. Furthermore, Sumiyoshi et al. (2014) examined gray matter changes in response to a period as short as 1 week of voluntary wheel-running. Analyses revealed gray matter changes in widely distributed regions of the cerebral cortex, including motor, somatosensory, association and visual areas but not the hippocampus or prefrontal cortex. Structural changes were kept up for a period of at least 1 week and correlated positive with the total running distance. Collectively, these results indicate that exercise-induced structural gray matter plasticity may shift from sensorimotor to prefrontal and limbic regions during the time course of physical exercise. Interestingly, such a shift from sensorimotor to prefrontal and limbic structural plasticity has already been observed during the course of practice of a complex whole-body balance task (Taubert et al., 2010, 2012; Sehm et al., 2014).

Below the cortical sheath, white matter tracts interconnect distant cortical regions to allow information processing within large-scale networks (Fields, 2008). In addition to changes in gray matter, aerobic fitness in cross-sectional studies as well as endurance exercise interventions have shown to affect white matter tract structure as well (Voss et al., 2013a; Chaddock-Heyman et al., 2014; Herting et al., 2014). A longitudinal study involving 33 patients with schizophrenia and 48 healthy controls (age 18–48 years, 60 males/21 females) randomly assigned the subjects to either a physical exercise or control condition (Svatkova et al., 2015). The intervention lasted 6 months and contained 1 h training sessions conducted twice weekly. The proportion of aerobic (for instance cycling, rowing, treadmill running) to anaerobic exercises (weight-based strengthening exercises) was 2:1. Using diffusion-tensor imaging (DTI), a method that assesses the diffusion properties of water molecules to infer microstructural white matter changes, Svatkova et al. (2015) found that 6 months of exercise training alters white matter microstructure specifically in fiber tracts implicated in motor functioning such as the corpus callosum, corticospinal tract and superior longitudinal fascicle. This effect was comparable for patients and healthy subjects.

Taken together, the aforementioned studies demonstrate that endurance exercise leads to structural adaptations in motor-related brain regions and associated fiber connections. Nonetheless, longitudinal MRI studies examining the relation between exercise-induced brain changes and subsequent motor learning-induced brain changes were not conducted yet. Furthermore, conclusions about the practical significance of macroscopic brain changes are hindered since the MRI-observable changes could be driven by very different cellular changes (Zatorre et al., 2012). To gain more insight about that, the next section will focus on neurobiological adaptations on a more fine-grained level of observation.

### Cellular Level

As already mentioned, motor learning is associated with changes in synaptic efficacy (LTP/LTD) (Sanes and Donoghue, 2000; Lee et al., 2014) which depends on structural changes at the synaptic level (Toni et al., 1999) and is linked to changes in the size of movement representations in M1. In contrast to motor learning, Kleim et al. (2002a) showed that endurance exercise (wheel running) did not alter forelimb movement representations which is in line with earlier findings that fail to show synaptic structural changes (e.g., synaptogenesis) in response to endurance exercise (Black et al., 1990) but instead a greater density of blood vessels in layer V of the forelimb motor cortex (Kleim et al., 2002a). Also, exercise-induced blood vessel density increases were reported in other rodent studies using histological methods (Black et al., 1990; Isaacs et al., 1992) as well as MRI (Swain et al., 2003). Thus, endurance exercise does likely not lead to neuronal adaptations (except of neurogenesis in the hippocampus) but exercise-induced vascular changes might contribute to subsequent learning-related neuroplasticity (Adkins et al., 2006) since memory formation and consolidation are energy-demanding processes (Tononi and Cirelli, 2014). Thus, an improved supply of oxygen and other fuels to motor regions might be of relevance.

### Molecular Level

#### BNDF and Lactate

On a molecular level, a concerted action of key neurochemicals is required for the occurrence of motor learning- and exercise-related physiological and structural changes (Korte et al., 1995; Boulanger and Poo, 1999; Monfils et al., 2005; He et al., 2013). Acute endurance exercise has been shown to enhance the levels of many memory-related trophic factors like BDNF, VEGF, and IGF or neuromodulatory transmitters like dopamine, epinephrine or norepinephrine in peripheral blood circulation (Rojas Vega et al., 2006; Rojas Vega et al., 2012a; Phillips et al., 2014).

Among the abovementioned neuromodulators and neurotrophic factors, BDNF is likely the best investigated and maybe the most important one. Using BDNF-mutant mice, Korte et al. (1995) first demonstrated that BDNF contributes to LTP expression. In the same year, it was reported that rats exposed to 7 days of voluntary wheel running exercise showed increased BDNF gene expression in the hippocampus and certain layers of the caudal neocortex (Neeper et al., 1995), providing first evidence that growth factors may be responsible for the beneficial effects of exercise on cognition and the brain. These observations led to a series of studies examining the effects of exercise on growth factor signaling, brain structure and function (Voss et al., 2013b).

BDNF is involved in all steps of memory formation from neuronal excitation to the induction and maintenance of early and late forms of LTP (Korte et al., 1995; Vaynman et al., 2003; Bekinschtein et al., 2008; Gómez-Pinilla and Feng, 2012). Importantly, this not only applies for the hippocampus but also for the motor system (He et al., 2013). BDNF and its receptor TrKB are important molecular intersections of exercise and motor learning (Klintsova et al., 2004).

Because the exogenous administration of BDNF is problematic in humans (for discussion see Fumagalli et al., 2006), natural ways to elevate BDNF levels and other neurochemicals are a promising way to counteract motor dysfunction and to enhance motor learning in healthy people. In this respect, intrahippocampal injection of BDNF enhances cognitive learning in mice (Alonso et al., 2002) and acute exercise-induced increases in peripheral BDNF levels correlate with behavioral parameters of motor skill learning (Skriver et al., 2014) although the exact relationship between central and peripheral BDNF is unclear (Di Lazzaro et al., 2007). Knowing that values of BDNF as well as other trophic factors and neuromodulatory transmitters typically increase through endurance exercise (Knaepen et al., 2010; Skriver et al., 2014) indicates that exercise may represent a promising and natural enhancement strategy for key factors involved in motor learning. It is unclear how long BDNF levels remain elevated after the exercise session. In general, exercise-induced increases in peripheral BDNF return to baseline levels within several minutes after cessation of exercise (Rojas Vega et al., 2006). However, animal research provides evidence for elevated cortical BDNF levels 5 h after completion of exercise, with the 5 h values exceeding those obtained immediately after exercise (Takimoto and Hamada, 2014). In contrast to this, many human studies examining BDNF levels in the resting state after a long-term exercise intervention report just small increases of circulating BDNF levels (Rojas Vega et al., 2012a; Szuhany et al., 2015), whereas higher exercise intensities might elicit a more pronounced BDNF increase (Baker et al., 2010). Furthermore, regular exercise may also enhance the BDNF response to an acute bout of exercise (Szuhany et al., 2015). Noticeably, a cross-sectional study examining the link between habitual physical activity and resting BDNF levels report even a negative correlation (Currie et al., 2009). This discrepancy might be explained by an increased BDNF clearance and uptake in other tissues like the brain (Knaepen et al., 2010; Rojas Vega et al., 2012a) as well as the different ways of how peripheral blood was analyzed for BDNF. Peripheral BDNF values are significantly influenced by analysis kits and BDNF determination in blood plasma, serum or whole-blood (Knaepen et al., 2010; Klein et al., 2011). While exercise immediately increases BDNF levels in the brain and periphery, their dwell time remains speculative.

Besides the changes in neurochemicals, exercise influences the energy supply of the brain. For example, recent investigations highlighted that lactate, elevated in response to exercise-induced anaerobiosis in the muscle cells (Robergs et al., 2004), is increasingly used as energy source for the brain and becomes the preferred fuel if arterial lactate values exceed the lactate values in the brain (Dalsgaard et al., 2004; Kemppainen et al., 2005; Boumezbeur et al., 2010). This fact is of particular importance since high lactate levels increased motor cortex excitability (Coco et al., 2010). Moreover, the availability of lactate plays a crucial role in long-term memory formation because the blockade of the expression of monocarbocylate-transporters (MCT), which catalyze the diffusion of lactate, reduces the transfer of lactate to astrocytes and neurons *in vitro* and impairs long-term memory in rats (Suzuki et al., 2011). Given this, the finding that an acute bout of exercise near or above the lactate threshold results in an elevated expression of MCT’s is potentially relevant (Takimoto and Hamada, 2014). However, it remains to be determined how regular exercise affects brain energetics and whether this might relate to motor function and memory. Maybe most important, lactate is directly involved in growth-factor signaling in response to exercise.

## Behavioral Evidence

This section reviews studies involving chronic or long-term endurance exercise and studies involving acute exercise to enhance motor learning. In contrast to cognition and declarative memory, only few studies have been published examining endurance exercise-induced improvements in motor learning. Acute protocols comprise endurance exercise activities on a single day that are intense enough to evoke a systemic physiological response. Typically, acute exercise takes place immediately before (think of classical warm-up) or immediately after a motor skill practice session. Long-term exercise includes studies examining the effects of endurance exercise over longer time periods (days, weeks, months) before motor skill learning. While both types of interventions have certain neurobiological mechanisms in common, they represent disparate strategies to affect memory. In general, long-term exercise aspires to enhance the responsiveness of the brain to new environmental stimuli through enhancement of learning-induced neuroplasticity. While this is also true for acute exercise prior to motor skill practice, this type of exercise additionally targets an optimal preparation for high performance in the upcoming training session, for example by increasing arousal. On the contrary, exercising after a practice session selectively impacts motor memory consolidation (Snigdha et al., 2014). This is especially relevant from a research-methodological perspective, since the effects of acute exercise likely outlast the practice session, thus not just affecting acquisition, but also consolidation (Roig et al., 2013). Therefore, the effects of acute exercise depend on its temporal positioning in relation to motor skill practice (Roig et al., 2012). Note that it is not possible in all cases to draw conclusions on motor learning defined as relatively permanent changes in motor behavior, since many studies lack delayed retention tests (Kantak and Winstein, 2012; Schmidt and Lee, 2014).

### Acute Exercise Before Learning

Generally, warm-up aims to prepare the central nervous, neuromuscular, cardiovascular and respiratory systems for the upcoming training session and therefore ensures high performance and reduction of injury risk (Shellock and Prentice, 1985). If training sessions target motor learning, the conditions for memory encoding should be optimized as well. From a psychological perspective, this may be induced by an optimal level of arousal that is in turn dependent on the nature of the task to be practiced (Schmidt and Lee, 2014). Likely, increased arousal is enabled by an exercise-induced elevation of catecholamines like dopamine, epinephrine or norepinephrine (Winter et al., 2007; Skriver et al., 2014). Additionally, as stated in the previous section, an upregulation of neurotrophic factors like BDNF may benefit subsequent learning-induced synaptic plasticity (Winter et al., 2007; Mang et al., 2014; Skriver et al., 2014). Furthermore, endurance exercise has shown to alter cerebral blood flow (Ogoh and Ainslie, 2009; Dietrich and Audiffren, 2011), reduce intracortical inhibition in exercised (Yamaguchi et al., 2012) as well as non-exercised limbs (Singh et al., 2014a; Smith et al., 2014) and to improve the conditions for the induction of synaptic plasticity (McDonnell et al., 2013; Mang et al., 2014; Singh et al., 2014b). What is the behavioral evidence with reference to the effects of acute endurance exercise prior to motor skill performance and learning?

One study specifically examined the role of acute exercise on motor skill acquisition and long-term motor memory (Roig et al., 2012). In an experimental design with 48 healthy young male subjects split into three groups, interval cycling was conducted either before (PRE) or after learning (POST) a visuomotor tracking task, whereas controls (CON) rested. The dependent variable was the absolute retention performance of a visuomotor tracking task (RMSE) after 1 h, 24 h, and 7 days. While no between-group differences regarding the rate of motor skill acquisition were registered, it was found that both exercise groups showed better retention compared with controls 24 h and 7 days after practice. The same working group published an association study correlating the peripheral blood plasma levels of several biomarkers with skill acquisition and retention of the tracking task (Skriver et al., 2014). Blood samples were drawn immediately after exercise (PRE condition as introduced above) or rest (CON). Interestingly, lactate (*r* = 0.877) and norepinephrine (*r* = 0.636) were associated with an improved rate of skill acquisition during practice. For skill retention 7 days after acquisition, correlations were found for norepinephrine levels in PRE (*r* = −0.584), with noticeable trends toward significance for earlier retention time points (1, 24 h). Likewise, plasma BDNF levels were associated with improved skill retention 1 h (*r* = −0.672) and 7 days (*r* = −0.608) after practice. An intriguing finding of Skriver’s study is the significant correlation of lactate with better skill retention at all measurement points (1 h: *r* = −0.658, 24 h: *r* = −0.715, 7 days: *r* = −0.672). We will discuss the potential role of lactate in motor learning more detailed in the next section (see Hypothetical Mechanisms for Exercise-Induced Improvements in Motor Learning). In controls (CON), none of the examined biomarkers correlated with neither skill acquisition nor retention with the exception of norepinephrine, which showed, somewhat surprisingly, the opposite pattern as observed in PRE, since higher blood plasma values at each measurement point indicated higher error values at skill retention (1 h: *r* = 0.530, 24 h: *r* = 0.535, 7 days: *r* = 0.529). Significant associations with skill acquisition and retention were not found for dopamine, IGF-1 and VEGF in neither group.

Inspired by Roig’s study, Mang et al. (2014) examined the effects of an acute bout of high-intensity exercise on PAS-induced LTP-like plasticity and on learning of an implicit visuomotor tracking task. A motor tracking task had to be acquired under different conditions and memorized approximately 24 h later. Subjects received either exercise or a resting control period before acquisition of a learning sequence. Serum BDNF blood samples were collected immediately before and after exercise. While the spatial task component of the tracking task was not affected by exercise, the temporal components improved from early to late practice and this improvement was preserved 24 h after practice in the exercise condition. Given the exercise-induced improvement especially of the timing-related task component, the authors hypothesized that exercise specifically affected cerebellar function (Mang et al., 2014). Despite the marked 3.4-fold increase in serum BDNF following exercise, significant correlations between normalized BDNF change and any behavioral data were not found. Note that a positive effect of an acute bout of exercise on skill acquisition was also observed by Statton et al. (2015). Using 30 min of moderate-intensity exercise prior to motor practice of a sequential motor task, Statton et al. (2015) observed improvements in skill acquisition but not skill retention which is in contrast to the above mentioned results of Roig et al. (2012) and may be induced by the different exercise intensities (high- vs. moderate-intensity).

As an intermediate result, the reported studies conducted in laboratory settings observed beneficial effects of an acute bout of high-intensity exercise prior to skill acquisition on motor learning as objectified with delayed retention tests (Roig et al., 2012; Mang et al., 2014) and enhanced performance improvements during initial practice (Statton et al., 2015). However, despite of the similar structure and intensity of exercise, a significant association of the behavioral data with BDNF was not consistently reported (Mang et al., 2014; Skriver et al., 2014).

Given the facts that the mastering of comparably simple skills like tracking does not require high amounts of practice and that it is a part-body movement questions the ecological validity of such findings, especially with respect to whole-body movements (Wulf and Shea, 2002). To gain insight into more complex motor learning processes, a recent meta-analysis focused exclusively on the performance of whole-body, psychomotor tasks following any type of moderate and strenuous acute conditioning exercise (endurance, resistance, balance) (McMorris et al., 2015). The results obtained from 28 studies involving 570 participants revealed a slightly positive effect size for moderate (*g* = 0.15), and a considerably negative effect size for high-intensity exercise (*g* = −0.86). These results are contrary to the view that moderate, and even more high-intensive, warm-up improves performance.

The reasons why especially resistance and high-intensity endurance exercise might have detrimental effects on performance are not examined systematically to date. Theoretically, this effect could be based on reduced cortical excitability (Takahashi et al., 2011) or increased intracortical inhibition (Sidhu et al., 2013). Notably, studies registering a positive effect of high-intensity exercise on motor learning used lower limb exercise to promote skills performed with the upper extremity (Roig et al., 2012; Mang et al., 2014). This suggests that a local peripheral and/or central fatigue mechanism may affect exclusively the pre-strained muscle groups, but not the non-exercised limbs (note that this might just apply for endurance and not for resistance exercise, c.f. Takahashi et al., 2011). In line with this, increased PAS-induced synaptic plasticity after 20 min high-intensity interval cycling was observed in the non-exercised abductor pollicis brevis muscle (Mang et al., 2014). Also remarkably, studies using low to moderate intensity endurance exercise showed facilitative effects on complex motor skill performance like shot putting (Anshel and Novak, 1989) or soccer dribbling (McMorris et al., 1994). This suggests that the facilitative effect of exercise prior to motor skill practice is effector-dependent and not limited to simple skills like tracking.

To sum up, evidence indicates that acute exercise improves motor skill learning but further research is required to disentangle the effector-specificity of this improvement. Based on the existing evidence, a negative effect on motor skill performance and learning might be expected if warm-up exercise is potentially fatiguing and involves at the same time the main effectors that are important for the execution of the skill to be practiced in succession.

### Acute Exercise After Learning

Immediately after motor practice, the motor memory trace is thought to be in a fragile state and practice-induced skill improvements need to be transformed into a persistent state (McGaugh, 2000; Robertson et al., 2004). This applies for both declarative and procedural memories (Mayford et al., 2012) and for the latter, incremental learning can be viewed as an ongoing cycle of consolidating fragile memory traces (Trempe and Proteau, 2012). This is relevant for the entire motor learning period because already stabilized memories may become partly labile through reactivation in a subsequent practice session, and thus need to be re-stabilized again (Alberini, 2005; Dudai, 2012).

While one promising possibility to facilitate consolidation is sleep, another lately discussed option might be a bout of endurance exercise immediately after practizing a motor skill. The theoretical basis of this strategy is that the neurobiological machinery of memory formation remains active after the termination of motor practice. In the first hours after practicing a motor skill, molecular blockade (Kleim et al., 2003) or downregulation of corticospinal excitability (Muellbacher et al., 2002) of M1 or learning a motor interference task (Brashers-Krug et al., 1996) can disrupt motor memory consolidation to a significant degree (reviewed in Robertson et al., 2004; Krakauer and Shadmehr, 2006). With the passage of time after initial practice cessation, the susceptibility to interferences gradually descends (Krakauer and Shadmehr, 2006).

From a neurobiological point of view, the persistence of LTP and its resistance against interfering stimuli could be the crucial mechanism allowing for proper skill consolidation (Cantarero et al., 2013). Intact BDNF release and function of its receptor TrkB are important for the persistence of LTP (Bekinschtein et al., 2008). Therefore, the exercise-induced elevation of neurotrophins like BDNF and catecholamines like norepinephrine (Segal et al., 2012; Skriver et al., 2014) might contribute to enhance offline learning and/or to minimize the effects of interfering stimuli in the consolidation time window.

As mentioned in the previous section, Roig et al. (2012) showed that acute high-intensity exercise immediately after skill acquisition facilitates long-term motor memory. When directly comparing the two intervention groups (exercised before [PRE] or after skill acquisition [POST]) it was shown that the group that exercised after practice outperformed the group that exercised before practice in the retention test 7 days after skill acquisition. Hence, this study provided first evidence that a single bout of exercise after practicing a motor skill can enhance off-line learning.

But does post-learning exercise also protect against task interference within the consolidation window? Rhee et al. (2015) asked undergraduate subjects to learn a motor sequence task. Three experimental groups practiced according to the classical memory stabilization paradigm: acquisition of the target sequence followed by practicing an alternative (interfering) sequence 2 h later and a retention test (consisting of three trials) of the target sequence 24 h after the first practice session. While one of these groups rested between acquisition of the target and alternative sequences (ALT), the experimental groups conducted an acute bout of exercise either immediately after the target sequence (IMM) or immediately before the alternative sequence (END). The authors found that exercise contributed to the emergence of an off-line performance gain in the retention test session despite of task interference. But this was only true for the first retention test trial in the END condition.

### Long-Term Exercise

Regular exercise training conducted over months or even years leads to numerous epigenetic adaptations in different organ systems and tissues including skeletal and cardiac muscle cells and the brain (Hawley et al., 2014; Heinonen et al., 2014). Recently, the use of long-term endurance exercise to prime the molecular machinery for subsequent learning is increasingly recognized by scientists from basic research (Berchtold et al., 2005, 2010; Korol et al., 2013). In line with this, long-term exercise before learning is assumed to be a promising intervention strategy especially for motor rehabilitation (Mang et al., 2013; Petzinger et al., 2013; Stoykov and Madhavan, 2015) suggesting a general positive transfer effect of endurance exercise on motor skill learning (Kleim and Jones, 2008) that has already been proved empirically (Quaney et al., 2009; Wang et al., 2015). However, there is a general lack of studies examining the effects of long-term exercise on motor learning and performance so that this area of research must be considered as largely underexplored to date (Stoykov and Madhavan, 2015).

A pilot study assessing the role of long-term physical activity on motor skill learning was conducted with 10 elderly subjects (age range 72–91 years) divided into two groups (Bakken et al., 2001). The exercise group passed through a physical activity program including calisthenics, stationary cycling and walking over 8 weeks (three training sessions/week), whereas controls rested. A finger-movement tracking task was tested before and after the 8 weeks. The exercise group showed a significant positive development in the accuracy index of a finger-movement tracking task from pre- to post-intervention compared with controls, whose performance worsened over time. However, the small sample size and the between-group differences especially regarding resting heart rate and blood pressure makes a generalization of the results difficult even for this age group.

In a more recent animal study, Buitrago et al. (2004) introduced the rotarod motor learning paradigm (balancing on an accelerated stick) and provided five rats daily access to a closed running wheel for a period of 7 days. The rats were kept in the wheel until they ran a predetermined distance of 100 m per day (except for day 1). Wheel-running was followed by 8 days of rotarod training. In the control condition, five rats exclusively practiced the rotarod task. Interestingly, the exercise group showed higher initial levels of rotarod performance and this advantage remained stable until the end of the rotarod training period. The authors interpreted this finding as a positive transfer effect of wheel-running movements to the rotarod task by means of an improved motor control through placement of steps to maintain balance and speed. However, one might counter the assumption that wheel running led to a specific transfer effect (for example, on balance ability) since running is considered to be a simple, well-practiced, automated and therefore hardly challenging movement skill for mice (Black et al., 1990). In line with this assumption, prior studies failed to observe synaptogenesis in response to wheel running (Black et al., 1990). The occurrence of a general positive transfer effect evoked by long-term exercise (Adkins et al., 2006; Kleim and Jones, 2008) should at least be considered as an alternative hypothesis to the assumption of a specific transfer of wheel running on locomotion-related abilities like balance.

The (sparse) existing evidence suggests that even comparably short periods of exercise are sufficient to prime the underlying neurobiological substrates for motor learning. Whether regular exercise over several months or years reveals additional benefits for motor learning is purely speculative to date. While a minimum amount of exercise is required to prime the molecular machinery for learning (Berchtold et al., 2005), the sustainability of exercise-induced adaptations is likely higher in the case of long-term compared with short-term exercise periods (Hötting and Röder, 2013).

## Hypothetical Mechanisms for Exercise-Induced Improvements in Motor Learning

**FIGURE 1:**
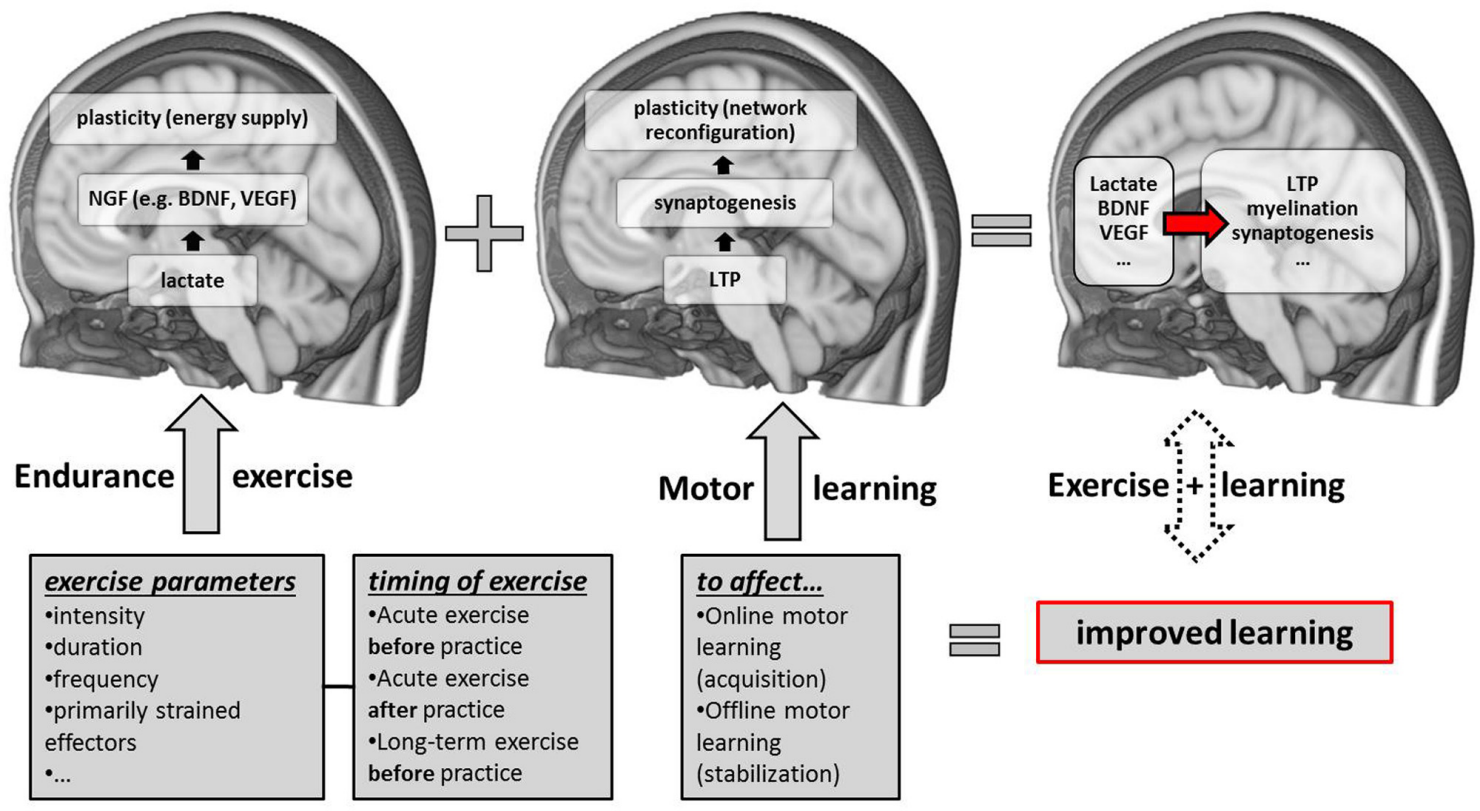
**Schematic overview of candidate neurobiological correlates and contributing factors (exercise parameters and the timing of exercise sessions with respect to motor practice) of exercise-induced improvements in motor learning.** NGF, nerve-growth factors; LTP, long-term potentiation; BDNF, brain-derived neurotrophic factor; VEGF, vascular-endothelial growth factor.

Our working hypothesis is that endurance exercise improves motor learning through facilitation of motor learning-related neuroplasticity (**Figure 1**). However, the causal link between exercise- and motor learning-related neuroplasticity has not yet been established (see Introduction). We previously reviewed behavioral and neurobiological evidence obtained in separate studies and we will now continue with the development of hypotheses concerning their mechanistic link.

At the molecular level, skeletal muscles can act as endocrine organs capable of secreting molecules relevant for neuroplasticity (Phillips et al., 2014; Lucas et al., 2015). Understanding the link between exercise-induced changes in peripheral biomarkers and the brain is of critical importance. Here, solid correlations between brain tissue and peripheral BDNF levels were found (Karege et al., 2002; Klein et al., 2011). A possible way by which exercise increases BDNF under physiological conditions could be the transport of peripheral-derived BDNF to the brain via the blood-brain-barrier (Pan et al., 1998; Di Lazzaro et al., 2007). However, Matthews et al. (2009) showed that BDNF mRNA and protein are increased in skeletal muscles after exercise, but the increased BDNF seems not be released into circulation. Analyses of blood samples from the radial artery and the internal jugular vein under resting and exercise conditions indicate that the brain itself may account for 70–80% of the BDNF levels circulating in peripheral blood vessels (Rasmussen et al., 2009). Therefore, changes in peripheral BDNF levels seem to be mainly caused by alterations in brain BDNF release into circulation.

A biomarker of potential interest for motor learning-induced neuroplasticity is lactate. Lactate is released from skeletal muscles during exercise and lactate in brain tissue modulates several brain functions (for overview see Barros, 2013) such as the survival of neurons (Fünfschilling et al., 2012; Lee et al., 2012) and axonal myelination (Rinholm et al., 2011). As we have outlined in the previous section, peripheral-derived lactate contributes significantly to brain metabolism under the conditions of physical exercise (van Hall et al., 2009; Boumezbeur et al., 2010). Also, lactate is assumed to play a major role in the exercise-induced elevation of neural growth factors. The link between lactate and growth factors is supported by studies that mimicked endurance exercise by sodium lactate injections. For example, Coco et al. (2013) treated cultures of astrocytes and SH-SY5Y (a cell line used as a model for neurons) *in vitro* for a period of 4 or 24 h with sodium lactate concentrations ranging from 5 to 25 mmol^∗^l^−1^. The results show that BDNF mRNA in the treated cultures is markedly increased in comparison to control cultures. When lactate was applied for 4 h, the BDNF mRNA increase was positively related to the concentration of sodium lactate in both cultures. This applied also for astrocytes after the 24 h treatment but not for the SH-SY5Y cells, where BDNF mRNA levels after 24 h returned to baseline. However, the exact mechanisms by which lactate increases BDNF mRNA remain to be clarified (Bergersen, 2015). In another *in vivo* study, Lezi et al. (2013) reproduced certain endurance exercise-related effects by infusing sodium lactate in resting mice. One of the main findings of this study is a lactate-induced elevation of VEGF levels, another neuroplasticity-related growth factor in the brain. Importantly, Schiffer et al. (2011) recently showed that the peripheral infusion of sodium lactate enhanced levels of serum BDNF in humans in the resting-state. Since sodium lactate has a basic pH-value, it is likely that increasing lactate concentrations instead of acidosis are causally linked with the observed changes in BDNF. In line with this, pH buffering via bicarbonate infusion during high-intensity cycling does not abolish the BDNF response, providing additional evidence that the exercise-induced elevation in BDNF-levels is indeed due to increased lactatemia (Rojas Vega et al., 2012b). Furthermore, it was found that lactate stimulates the expression of genes required for long-term memory *in vitro* and *in vivo* (Yang et al., 2014). To sum up, at the molecular level, studies indicate a positive relationship between lactate levels and the concentration of neurotrophic factors, especially BDNF (Ferris et al., 2007), with strong evidence that this relationship may be causal in nature (Schiffer et al., 2011; Coco et al., 2013; Lezi et al., 2013). Despite of the absence of a correlation between exercise-induced elevations of lactate and BDNF levels after cessation of exercise, Skriver et al. (2014) showed that both biomarkers *per se* were highly associated with successful motor skill learning.

How can exercise regimens be improved to optimize neuroplasticity? The aforementioned studies indicate the importance of high exercise intensities for a high BDNF response (Knaepen et al., 2010; Huang et al., 2014), which may be mediated by an exercise-induced increase of lactate levels. Beyond that, high exercise intensities are proposed to increase cardiovascular health (Lucas et al., 2015) and showed beneficial effects on various cognitive functions (Angevaren et al., 2007; Ferris et al., 2007; Winter et al., 2007) and motor learning (Roig et al., 2012; Mang et al., 2014).

Exercise interventions that elevate peripheral BDNF levels include ramp or graded exercise tests to exhaustion (Rojas Vega et al., 2006, 2012a), continuous exercise of moderate to high intensities (Gold et al., 2003; Ferris et al., 2007; Schmidt-Kassow et al., 2012; Schmolesky et al., 2013) and high-intensity interval (HIIT) as well as sprint interval training (Winter et al., 2007; Mang et al., 2014; Skriver et al., 2014). In contrast to ramp exercise and continuous exercise, interval training consists of repeated bouts of exercise interspersed with recovery periods that comprise light exercise or rest (Billat, 2001) and is considered as an effective training method to improve endurance ability (Milanović, 2015). Moreover, as shown in animal research, 6 weeks of endurance training (six times weekly) with either HIIT (95–100% VO_2_max) or continuous exercise (80% VO_2_max) elevated BDNF and GDNF (glial cell line-derived neurotrophic factor) in rat brain tissue in comparison to a resting control group (Afzalpour et al., 2015). Moreover, the HIIT condition led to significantly higher BDNF and GDNF levels compared with the continuous condition (Afzalpour et al., 2015). The reason for the superiority of HIIT might be that HIIT training can be performed at velocities above the individual anaerobic threshold (IAT) (Billat, 2001), therefore allowing to subsequently accumulate considerable levels of lactate (Buchheit and Laursen, 2013). On the contrary, continuous endurance exercise over longer durations have to be performed at intensities low enough *not* to induce lactate accumulation above the IAT to avoid fatigue (Rojas Vega et al., 2012b).

However, an important and unresolved issue to date is whether an exercise intervention should affect either the peak BDNF level at a fixed time point, for example after the cessation of exercise, or the total volume of circulating BDNF over time (Schmolesky et al., 2013). To make matters worse, the kinetics of exercise-induced BDNF changes during training are largely unknown to date, but existing data suggest that BDNF values reach their maximum level after approximately 10–20 min of moderate intensive continuous exercise and show a slight decrease thereafter (Schmidt-Kassow et al., 2012). Nonetheless, long-term exercise interventions aiming at priming the molecular machinery of motor skill acquisition and stabilization might be most effective when conducted with high intensities.

Of note, the exercise-effects on motor learning may also be dependent on the nature of the motor task (Wulf and Shea, 2002) because the brain networks involved in early and late practice depend on task complexity. Knowledge of the brain regions being involved in different stages of skill learning is critical to optimize exercise schedules that influence motor skill acquisition, consolidation and retention. Therefore, future studies are required that combine exercise and (subsequent) motor learning with observation of underlying brain changes. Disentangling the brain regions that correlate with the exercise-induced improvement in motor learning is critical to subsequently prove causality with, for example, focal brain stimulation (e.g., TMS).

Notwithstanding, recommendations regarding optimal exercise regimens are even more difficult to provide if motor skill learning should be affected by an acute bout of exercise. Even though some studies present evidence for a beneficial effect of HIIT on motor skill learning (Roig et al., 2012; Mang et al., 2014), this benefit might not apply for complex motor skill learning (McMorris et al., 2015). In the case of acute exercise prior to motor skill practice, reduced motor performance might be due to temporary peripheral and/or central fatigue effects (Taylor, 2012), especially relevant if the pre-strained effectors are at the same time critically involved in the performance of the motor skill. Besides increasing intracortical inhibition of pre-strained muscles (Sidhu et al., 2013), high exercise intensities are also known to enhance cortisol levels (Rojas Vega et al., 2006). Since low-to-moderate exercise intensities mainly revealed facilitating effects on various neuroplasticity indices and behavior, these intensities can be recommended for applied settings at the moment. However, high-intensity exercise might be useful if part-body movements of the upper limb should be facilitated by lower limb exercise (Roig et al., 2012; Mang et al., 2014) and maybe vice versa.

On the contrary, temporary fatigue effects theoretically should not be of disadvantage if exercise is conducted after practicing motor skills (Roig et al., 2012). However, further research is needed because the neuronal mechanisms that mediate motor memory consolidation in the time window after practice are not known well by now (Berghuis et al., 2015), let alone their potential interaction with a post-practice bout of exercise.

To conclude, considerable knowledge gaps remain regarding the optimal type, intensity, duration and, if applicable, frequency of exercise to promote motor learning related neuroplasticity (van Praag et al., 2014). However, especially the results obtained from basic research lay the foundation for more applied studies to be conducted in the future. In our view, properly scheduled endurance exercise protocols potentially reflect a promising intervention strategy to affect motor learning.

## Author Contributions

All authors (MT, AV, NL) discussed the material presented in this review and wrote the manuscript.

## Conflict of Interest Statement

The authors declare that the research was conducted in the absence of any commercial or financial relationships that could be construed as a potential conflict of interest.
